# Self-referenced method for the Judd–Ofelt parametrisation of the Eu^3+^ excitation spectrum

**DOI:** 10.1038/s41598-021-04651-4

**Published:** 2022-01-12

**Authors:** Aleksandar Ćirić, Łukasz Marciniak, Miroslav D. Dramićanin

**Affiliations:** 1 Vinča Institute of Nuclear Sciences, National Institute of the Republic of Serbia, University of Belgrade, P.O. Box 522, 11001 Belgrade, Serbia; 2 Institute of Low Temperature and Structure Research, PAS, ul. Okólna 2, 50-422 Wrocław, Poland

**Keywords:** Optics and photonics, Chemical physics, Optical physics, Atom optics

## Abstract

Judd–Ofelt theory is a cornerstone of lanthanides’ spectroscopy given that it describes 4f^n^ emissions and absorptions of lanthanide ions using only three intensity parameters. A self-referenced technique for computing Judd–Ofelt intensity parameters from the excitation spectra of Eu^3+^-activated luminescent materials is presented in this study along with an explanation of the parametrisation procedure and free user-friendly web application. It uses the integrated intensities of the ^7^F_0_ → ^5^D_2_, ^7^F_0_ → ^5^D_4_, and ^7^F_0_ → ^5^L_6_ transitions in the excitation spectrum for estimation and the integrated intensity of the ^7^F_0_ → ^5^D_1_ magnetic dipole transition for calibration. This approach facilitates an effortless derivation of the Ω_6_ intensity parameter, which is challenging to compute precisely by Krupke’s parametrisation of the emission spectrum and, therefore, often omitted in published research papers. Compared to the parametrisation of absorption spectra, the described method is more accurate, can be applied to any material form, and requires a single excitation spectrum.

## Introduction

Lanthanides have revolutionised the modern science and technology and are present in almost any device^1^. The global value of lanthanide-containing products estimated in 2014 was 1.5–2 trillion dollars^2^, and this number has been continuously increasing since that time. Moreover, the use of lanthanides in phosphors accounts for approximately 3% of the total market share^1^. Considering lanthanide applications in phosphors, researchers focus on luminescent properties, which make these compounds unique among other luminescence centres. Owing to the characteristic electronic configuration of trivalent lanthanide ions, their luminescence due to *4f–4f* electronic transitions is characterised by the narrow emission and absorption bands, host-independent transition energies, and plethora of emissions spanning across the ultraviolet–near infrared (NIR) spectral range with long emission decays and high quantum efficiencies^3^.

From the viewpoint of the quantum theory developed in the 1920–1930s, the spectral properties of lanthanides were puzzling as summarised by Van Vleck in his famous article ‘The Puzzle of Rare-earth Spectra in Solids’ published in 1937^4^. In particular, the high intensities of intra-configurational 4f transitions contradicted the parity (Laporte) selection rule^5^. Owing to development of Racah’s algebra in 1949 and first computers allowing the tabulation of many required coefficients, two equivalent articles were published almost simultaneously in 1962 by Judd in Physical Review^6^ and Ofelt in The Journal of Chemical Physics^7^ which were characterised by B. Wybourne in the following words^8^:

‘The two papers of 1962 represent the paradigm that has dominated all future work…up to the present time’

What was later coined as the Judd–Ofelt theory (JO) provided the first quantum–mechanical explanation of the intensities of induced electric dipole transitions. The centrepiece of this theory includes three intensity parameters Ω_λ_, λ = 2, 4, 6, from which many ‘derived quantities’ with high practical importance (such as radiative transition probabilities, radiation lifetimes, branching ratios, cross-sections, and intrinsic quantum yields) can be simply obtained. These parameters may be subsequently used to calculate the intensities of the entire emission or absorption spectra^9^.

**Figure 1:**
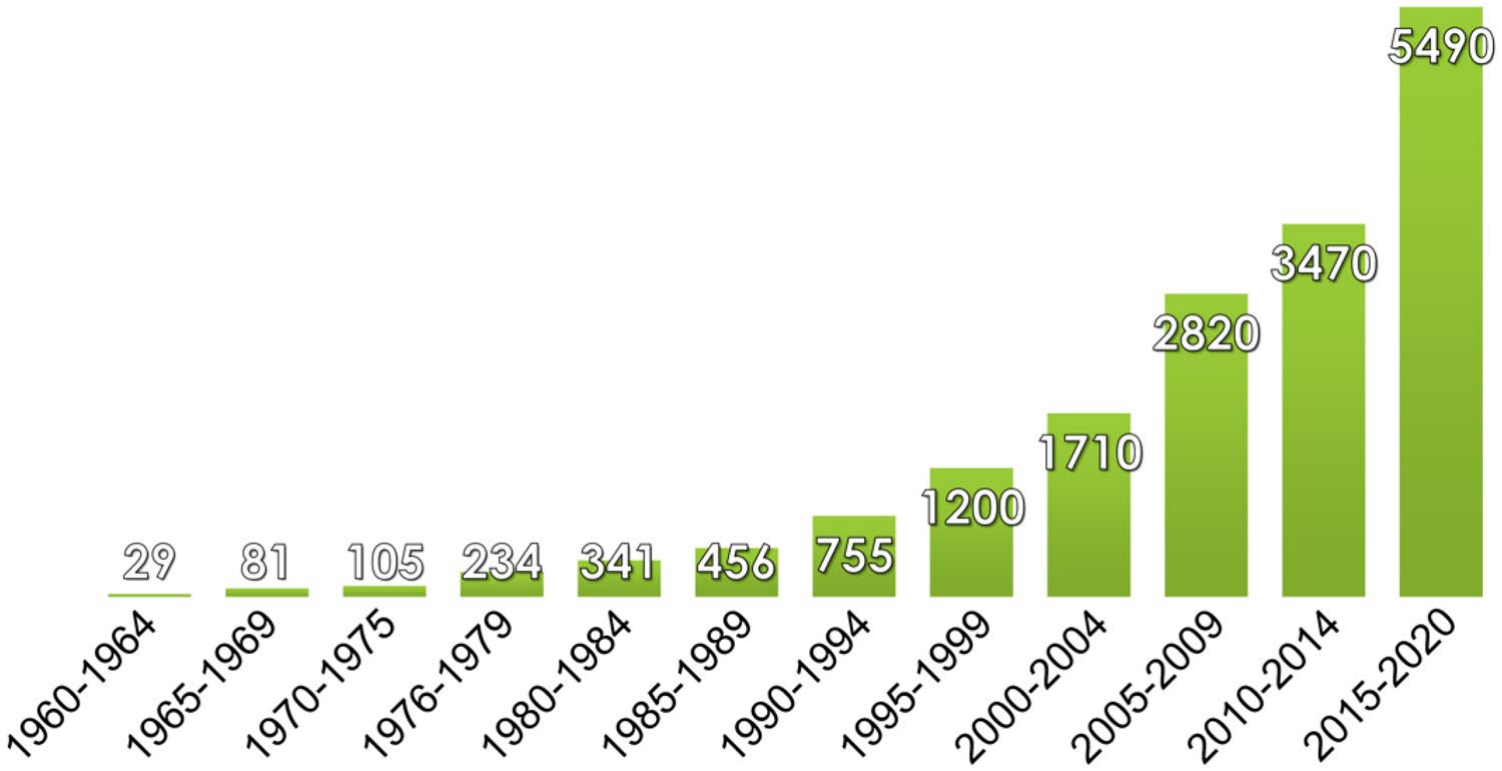
Numbers of published papers with the expression “Judd–Ofelt” determined for 5-year intervals by Google Scholar (accessed in July 2021).

Considering the capability of the JO theory and spectroscopic importance of lanthanides, it is not surprising that the research interest in this theory is rapidly growing (Fig. 1).

The ongoing research studies in this field can be classified into three categories: (i) theory improvement and development of alternate JO parametrisation methods^5,10–17^, (ii) JO parametrisation of lanthanides in different hosts doped at various concentrations and analysis of lanthanide-activated phosphors synthesised by different methods (see Tables 10–21 in Ref.^18^), and (iii) applications of JO theory and JO parameters by constructing various models in other areas of science related to luminescence^19,20^. The majority of research works on these topics fall into categories (ii) and (iii), demonstrating that the practical implementation of the JO theory does not require its deep understanding. For category (ii), it is necessary to know the methods for calculating JO parameters, while for research category (iii), such parameters can be obtained from the literature.

## Previous JO parametrisation studies

JO parameters are traditionally determined by analysing the absorption spectra of trivalent lanthanide-activated materials. This method is described in detail in Refs.^5,9^; therefore, only its brief description is provided below. It is based on fitting the experimental oscillator strengths obtained from the absorption spectrum with theoretical equations derived for selected transitions of a given lanthanide ion. The experimental oscillator strength (*P*_*exp*_) is equal to^18^

1 $$\begin{array}{c}{P}_{\text{exp}}\left[\text{/}\right]=4.32\cdot \frac{{10}^{-9}\Xi }{{X}_{A}},\end{array}$$

where

2 $$\begin{array}{c}\Xi =\int \varepsilon \left(\nu \right)d\nu ,\end{array}$$

is the integrated molar absorptivity, $\nu$ is the wavenumber (cm^−1^), and *X*_*A*_ is the fractional thermal population at the initial level. ε (mol^−1^ L cm^−1^) is the molar extinction coefficient (molar absorptivity), which can be calculated from absorbance by the following formula:

3 $$\begin{array}{c}\varepsilon =Cd/A.\end{array}$$

Here, *C* (mol/L) is the concentration, and *d* (cm) is the length of the optical path in a given material.

At temperatures above the absolute zero, the higher-lying energy levels of lanthanide ions are thermally populated with probabilities specified by the Boltzmann distribution. If the energy separation to the next level is larger than 2000 cm^−1^, the thermalisation of the current energy level is not efficient and may not follow the Boltzmann distribution (in this case, it can be even neglected due to its low contribution). The ratio of the optical centres at a selected level to the total number of optical centres is represented by the fractional thermal population^18,21^:

4 $$\begin{array}{c}{X}_{A}=\frac{{g}_{A}\mathrm{exp}\left(-\frac{\Delta {E}_{A}}{kT}\right)}{\sum_{i}{g}_{i}\mathrm{exp}\left(-\frac{\Delta {E}_{i}}{kT}\right)},\end{array}$$

where *T* (K) is the temperature, *g*_*i*_ is the level of degeneracy, Δ*E*_*i*_ is the energy difference between level *i* and the ground state (in cm^−1^), and *k* = 0.695 cm^−1^·K^−1^·is the Boltzmann constant. According to the fractional thermal population of the Eu^3+^ ground multiplet ^7^F_J_, the ^7^F_1_ level is significantly populated even at room temperature due to the low energy separation between the ^7^F_0,1_ levels, which can be verified by the excitation or absorption spectra that contain transitions originating from the Eu^3+ 7^F_1_ level^22^.

Unlike the oscillator strength, dipole strength is independent of the photon energy and related to the oscillator strength via the following expression:

5 $$\begin{array}{c}{P}_{\text{th}}\left[\text{/}\right]=4.702\cdot {10}^{29} \tilde{\nu } {D}_{th},\end{array}$$

where $\tilde{\nu }$ is the transition barycentre energy (in cm^−1^), and *D*_th_ is the dipole strength. The dipole strength of the electric dipole (ED) transition is defined as^9^

6 $$\begin{array}{*{20}c} {D_{{th}}^{{ED}} \left[ {{\text{esu}}^{2} {\text{cm}}^{2} } \right] = e^{2} \sum\limits_{\lambda } {\Omega _{\lambda } } U^{\lambda } ,} \\ \end{array}$$

where U^λ^ are the squared reduced matrix elements (RMEs), and *e* = 4.803 × 10^10^ esu is the elementary charge. RMEs are often considered host-independent; for this reason, many researchers have consorted to using the values tabulated by Carnall et al.^23^ instead of calculating them for a particular host by employing Slater integrals and spin–orbit coupling parameters^24^. The magnetic dipole (MD) transition has a dipole strength that is also host-independent. The tabulated values for all MD transitions of all trivalent lanthanides are provided in Ref.^25^.

The experimental oscillator strength is compared to the theoretical strength by the formula

7 $$\begin{array}{c}{P}_{\text{exp}}=\frac{\chi }{g}{P}_{\text{th}},\end{array}$$

where *χ* is the local field correction, and *g* = 2 J + 1 is the degeneracy of the J level, from which the transition originates. The Lorenz field correction for the ED transition and local field correction for the MD transition during absorption are computed as follows^26^:

8 $$\begin{array}{c}{\chi }_{ED}^{ab}=\frac{{\left({n}^{2}+2\right)}^{2}}{9n}, { \chi }_{MD}^{ab}=n,\end{array}$$

where *n* is the wavelength-dependent refractive index. Ideally, the refractive index is calculated using the dispersion relation.

To obtain JO parameters, all *P*_exp_ magnitudes should be fitted to *P*_th_ using Eq. (7) for the observed transitions, thereby minimising the discrepancies between the theoretical and experimental values. Ultimately, this will produce Ω_λ_ values closest to the experimental data. As pointed out by Blasse and co-workers, a drawback of this method is the necessity to accurately measure the density of ions in the analysed sample. In addition, absorption intensity can be routinely measured only for glasses, transparent solutions, and crystals, leaving out non-transparent materials and crystalline powders^13^. Another drawback of the described approach is a high error of ~ 20%^27^ caused by the absence of higher-order contributions, whose inclusion significantly complicates the calculation procedure (see Ref.^28^). The third problem arises with the parametrisation of the Pr^3+^ ion as the proximity of the *4f5d* levels mixes with the *4f* levels, leading to a case that cannot be treated by the original JO method. As a result, complex alternative parameterisation methods with questionable accuracies were developed for Pr^3+^ ions^11^. Parametrisation using crystal field parameters is called ab initio parameterisation; however, it suffers from high complexity and limited accuracy, as stated by L. Smentek^10^:

‘Indeed, there are objective, or rather technical reasons, why it is still impossible to perform ab initio calculations that would provide reliable results.’

The readers interested in this method are referred to Refs.^18,29,30^.

Various techniques similar to the absorption-based one, which utilise excitation^15^ or diffuse-reflectance^31^ spectra, have been proposed in recent years. Their development was motivated by the limited application of the absorption method for powders and non-transparent materials. The methods are based on the comparison of the theoretical line strengths, *S*_calc_ = ΣΩ_λ_U^λ^, with the measured line strengths which are proportional to the peaks in the diffuse-reflectance or excitation spectrum. Although these techniques can be used for any material, they produce only relative JO parameters, which must be calibrated against the radiative transition probability of a selected level that is approximately equal to the inverse of the experimentally measured lifetime. By this spectrum calibration, the unknown parameter *c* vanishes, and the absolute values of the JO parameters are obtained. This assumption inherently introduces an error into the calculated values. For this reason, the authors of both the above-mentioned methods have chosen the first excited level of Er^3+^ for the calibration by the excited-level lifetime value (because it is almost purely radiative) and Nd^3+^ ion for the diffuse-reflectance method. Luo et al. have predicted that their excitation method parametrisation can be used on 10 lanthanide ions, among which is not the Eu^3+^ ion^15^. In recent years, this method has been tested on Dy^3+^ ion with success^32,33^.

Sytsma and Blasse were the first researchers who performed spectrum calibration using an excited-level lifetime for the JO parametrisation of the Gd^3+^ emission spectrum^13^ assuming that the deexcitation of its first excited level, which lied high above the ground level, was purely radiative. A similar approach was explored in our previous research study^12^ describing a JO parametrisation method that utilised the Pr^3+^ emission spectrum. Because the emissive ^3^P_0_ level is non-degenerate, parametrisation can be performed using the low-temperature emission spectrum with negligible temperature quenching. At low Pr^3+^ concentrations, the depopulation of the excited states through the interionic processes was very small, and the radiative lifetime of the ^3^P_0_ level was equal to the experimentally measured value. This allowed conducting more accurate spectrum calibration and JO parameterisation than the corresponding procedures of the alternative absorption methods mentioned above.

In 1966, shortly after Judd and Ofelt had published their articles, Krupke developed a JO parametrisation method using the emission spectrum of the Eu^3+^ ion^17^. Because this method includes the higher-order contributions not considered in the traditional parametrisation of the absorption spectrum, it remains the most accurate JO parameterisation technique. Unlike the methods that require calibration with the excited level lifetime, Krupke exploited the fact that Eu^3+^ had a pure host-independent MD transition ^5^D_0_ → ^7^F_1_, to which other intensities could be compared. In the method proposed in our previous work^16^, the pure MD ^5^D_1_ → ^7^F_0_ emission is used for spectral calibration and, as will be demonstrated later, the same energy levels are utilised in the novel parametrisation technique developed in this study. Owing to the use of an accurate dispersion relation for the refractive index, the JO parameterisation methods based on the emission spectra of the Eu^3+^ ion are unbeatable in terms of accuracy and simplicity. However, in such spectra, the only transition that can be used to calculate the Ω_6_ parameter lies in the NIR region outside the detection limits of most traditional detectors and is also very weak due to the low U^6^ = 0.0002 RME^34^. Only a limited number of studies have reported the ^5^D_0_ → ^7^F_6_ emission^35–39^. Thus, JO parameterisations performed using Eu^3+^ emission spectra are often incomplete. Furthermore, because U^6^ has a low value, it is estimated with relatively large error and variations^34,40^, making the parameterisation of the Ω_6_ value based on the emission spectrum unreliable and difficult to perform. Despite its low importance for Eu^3+^ emission, Ω_6_ is the most important parameter in the absorption/excitation spectrum, as the most intense absorption, ^7^F_0_ → ^5^L_6_, depends solely upon its value. The Ω_6_ magnitude is related to the rigidity of a medium where ions are incorporated^41,42^, which in turn depends on the Debye temperature^43,44^. Consequently, there is experimental and theoretical incompleteness of the JO parametrisation of Eu^3+^-activated materials that are not glasses, crystals, or solutions.

### Self-calibrated JO parametrisation of the Eu^3+^ excitation spectrum (JOEX)

Therefore, to perform accurate JO parameter determination and avoid the limitations of the methods described above, we propose a novel technique for the JO parametrisation of Eu^3+^-doped materials, which enables the estimation of all three Ω_λ_ parameters from a single excitation spectrum. Unlike the other methods that rely on spectrum calibration by the excited-level lifetime, this approach utilises the pure MD transition ^7^F_0_ → ^5^D_1_ at 525 nm for calibration purposes. The proposed method simultaneously facilitates the determination procedure and increases the reliability of the obtained results. The versatility of this technique allows its application to non-transparent and powder materials, for which other methods are not suitable. It includes all higher-order contributions to the JO parameters (the original absorption spectrum-based method utilised only a static coupling model)^45^. Owing to the high U^6^ value obtained for the ^7^F_0_ → ^5^L_6_ electronic transition of Eu^3+^ ions, U^6^ was used with low relative uncertainty. In addition, due the high intensity of the band associated with the ^7^F_0_ → ^5^L_6_ transition, its integrated intensity was also estimated with low uncertainty, contrary to the ^5^D_0_ → ^7^F_6_ emission in Krupke’s method.

In order to verify the reliability of the data obtained by the proposed method, the latter was applied to two different (from the chemical and morphological perspectives) materials: well-known Eu^3+^-activated Y_2_SiO_5_ microcrystalline phosphor and β-NaYF_4_ nanoparticles. The determined parameters were compared with the results obtained by Krupke’s parametrisation technique and the emission spectrum calculated by the JOES application software (https://omasgroup.org/joes-software/)^46^.

## Theoretical approach

The experimental dipole strength of a randomly oriented system (e.g., powder) in its absorption spectrum is equal to^18^.

9 $$\begin{array}{c}{D}_{\text{exp}}\left[{\text{esu}}^{2}{\text{cm}}^{2}\right]=\frac{\Xi }{108.9\cdot {10}^{36}\tilde{\nu }{X}_{A}}.\end{array}$$

The experimentally obtained dipole strength can be compared with the theoretical value using the formula.

10 $$\begin{array}{c}{D}_{\text{exp}}=\frac{\chi }{g}{D}_{\text{th}}.\end{array}$$

These equations are suitable for both ED and MD transitions; therefore, local field corrections must be applied accordingly. For pure MD and ED transitions, Eq. (10) can be modified as follows:

11 $$\begin{array}{c}\frac{{\Xi }_{\mathrm{MD}}}{108.9\cdot {10}^{36}{\tilde{\nu }}_{MD}{X}_{A}^{MD}}=\frac{{\chi }_{MD}}{{g}_{MD}}{D}_{\text{MD}}^{th},\end{array}$$

12 $$\begin{array}{*{20}c} {\frac{{\Xi _{{{\text{ED}}}} }}{{108.9 \cdot 10^{{36}} \tilde{\nu }_{{ED}} X_{A}^{{ED}} }} = \frac{{\chi _{{ED}} }}{{g_{{ED}} }}e^{2} \sum\limits_{\lambda } {\Omega _{\lambda } } U^{\lambda } .} \\ \end{array}$$

In the case of the *4f–4f* lanthanide transitions, the excitation spectrum is assumed to be identical to the corresponding absorption spectrum multiplied by a constant. Because absolute values cannot be obtained from excitation spectra, only $c{D}_{\text{exp}}$ may be calculated via Eq. (9) as follows:

13 $$\begin{array}{c}c{D}_{\text{exp}}\left[{\text{esu}}^{2}{\text{cm}}^{2}\right]=\frac{\Gamma }{108.9\cdot {10}^{36}\tilde{\nu }{X}_{A}},\end{array}$$

where Γ = *c*Ξ is the integrated intensity in the excitation spectrum for the corresponding transition, which is equal to the integrated molar absorptivity multiplied by the unknown *c* coefficient. Thus, the knowledge of *c* would allow JO parameterisation using Eq. (12). For the pure Eu^3+^ ED transitions ^7^F_0_ → ^5^D_4_ (λ = 4), ^7^F_0_ → ^5^L_6_ (λ = 6), and ^7^F_0_ → ^5^D_2_ (λ = 2), the theoretical dipole strength can be expressed as

14 $$\begin{array}{c}{D}_{\lambda }={e}^{2}{\Omega }_{\lambda }{U}^{\lambda },\end{array}$$

considering that all RMEs other than U^λ^ are equal to zero (see Table 1). In this case, Eq. (13) becomes

**Table 1:** RMEs of various transitions relevant for the JO parametrisation of the Eu^3+^ excitation spectrum.

Transition	U^2^	U^4^	U^6^
^7^F_0_ → ^5^D_2_	0.0009	0	0
^7^F_0_ → ^5^D_4_	0	0.0011	0
^7^F_0_ → ^5^L_6_	0	0	0.0153

15 $$\begin{array}{c}\frac{{\Gamma }_{\lambda }}{108.9\cdot {10}^{36}{\tilde{\nu }}_{\lambda }{X}_{A}\left({{}_{ }{}^{7}F}_{0}\right)}=c{\chi }_{\lambda }{e}^{2}{\Omega }_{\lambda }{U}^{\lambda }.\end{array}$$

Note that the degeneracy term is absent from this formula because J = 0 for the initial level.

In our recent article^16^, we exploited the pure MD emission ^5^D_1_ → ^7^F_0_ with an MD strength of 1.8 × 10^−42^ esu^2^ cm^2^. Because the dipole strength values determined for the emission and absorption/excitation processes are identical, the same dipole strength holds for the ^7^F_0_ → ^5^D_1_ transition (further abbreviated as *D*_MD_). As a result, Eq. (13) for the MD transition can be written in the following form:

16 $$\begin{array}{c}\frac{{\Gamma }_{MD}}{108.9\cdot {10}^{36}{\tilde{\nu }}_{MD}{X}_{A}\left({{}_{ }{}^{7}F}_{0}\right)}=c{\chi }_{MD}{D}_{MD}.\end{array}$$

After dividing Eq. (15) by Eq. (16), the fractional level populations vanish because the initial levels for calibrating the MD and ED transitions are the same. The unknown *c* parameter also disappears from the equation. As a result, a set of three equations for the determination of the JO parameters from the excitation spectrum is obtained:

17 $$\begin{array}{c}{\Omega }_{\lambda }\left[{\text{cm}}^{2}\right]=\frac{{\chi }_{MD}}{{\chi }_{\lambda }}\frac{{\tilde{\nu }}_{MD}}{{\tilde{\nu }}_{\lambda }}\frac{{D}_{MD}}{{e}^{2}{U}^{\lambda }}\frac{{\Gamma }_{\lambda }}{{\Gamma }_{MD}}, \quad \lambda =2, 4, 6.\end{array}$$

Note that a similar equation can be derived by using the ^7^F_1_ → ^5^D_0_ pure MD transition with a dipole strength 9.56 × 10^−42^ esu^2^ cm^2^^25^, but it would require the inclusion of degeneracies and fractional thermal populations.

## Parametrisation procedure

A straightforward algorithm for obtaining JO intensity parameters from the excitation spectrum of Eu^3+^ is outlined below.

1. The excitation spectra of Eu^3+^-activated materials can be obtained by monitoring the emission from the ^5^D_0_ level (from 350 to 550 nm). Monitoring the ^5^D_0_ → ^7^F_2_ emission at approximately 612 nm is the best option due to its high intensity. The emission from the ^5^D_0_ → ^7^F_4_ transition can be also used; however, one should be aware of the overlap with the second harmonic at 700 nm (for 350 nm excitation). The excitation band of the ^7^F_0_ → ^5^D_4_ transition is observed at approximately 364 nm, while that of ^7^F_0_ → ^5^L_6_ is detected at approximately 395 nm, ^7^F_0_ → ^5^D_2_—at 467 nm, and ^7^F_0_ → ^5^D_1_—at 525 nm.
2. The intensities of the excitation bands must be integrated, and their barycentres should be determined. It is easier to obtain the barycentre wavelength (sometimes called a centroid). In this case, the integrated intensities are equal to Γ_2_ = Γ(^7^F_0_ → ^5^D_2_), Γ_4_ = Γ(^7^F_0_ → ^5^D_4_), Γ_6_ = Γ(^7^F_0_ → ^5^L_6_), and Γ_MD_ = Γ(^7^F_0_ → ^5^D_1_). The wavelength barycentres are denoted by symbol $\tilde{\lambda }$. Owing to the nature of the Eu^3+^ excitation spectrum, they are almost equal to the wavelengths of the peak maxima. In some hosts the charge-transfer band may overlap with the ^7^F_0_ → ^5^D_4_, ^5^L_6_ excitations. Then the charge-transfer band must be subtracted by spectrum deconvolution prior to peak integration.
3. A refractive index value should be determined for each transition using the dispersion relation (if possible). For several hundred materials, dispersion relations are stored in a refractive index online database^47^. The values of the refractive index are computed by the formulas *n*_2_ = *n*(467 nm), *n*_4_ = n(364 nm), *n*_6_ = *n*(395 nm), and *n*_MD_ = *n*(525 nm).
4. The following three simplified equations can be used for parametrisation:
  18 $$\begin{array}{c}{\Omega }_{2}=7.803\frac{{n}_{2}{n}_{MD}}{{\left({n}_{2}^{2}+2\right)}^{2}} \frac{{\tilde{\lambda }}_{2}}{{\tilde{\lambda }}_{MD}}\frac{{\Gamma }_{2}}{{\Gamma }_{MD}}\cdot {10}^{-20}{\text{cm}}^{2},\end{array}$$
  19 $$\begin{array}{c}{\Omega }_{4}=6.384\frac{{n}_{4}{n}_{MD}}{{\left({n}_{4}^{2}+2\right)}^{2}} \frac{{\tilde{\lambda }}_{4}}{{\tilde{\lambda }}_{MD}}\frac{{\Gamma }_{4}}{{\Gamma }_{MD}}\cdot {10}^{-20}{\text{cm}}^{2},\end{array}$$
  20 $$\begin{array}{c}{\Omega }_{6}=0.459\frac{{n}_{6}{n}_{MD}}{{\left({n}_{6}^{2}+2\right)}^{2}} \frac{{\tilde{\lambda }}_{6}}{{\tilde{\lambda }}_{MD}}\frac{{\Gamma }_{6}}{{\Gamma }_{MD}}\cdot {10}^{-20}{\text{cm}}^{2}.\end{array}$$

**Figure 2:**
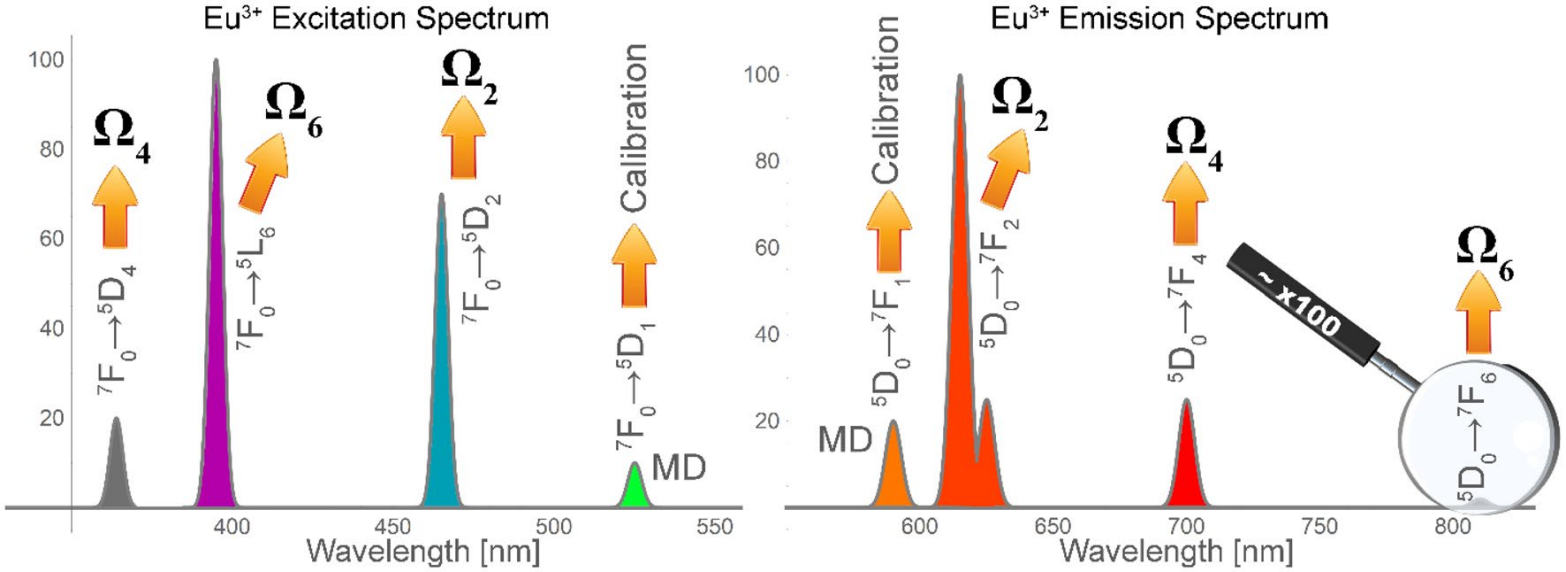
JO parametrisation schemes using the excitation and emission spectra of the Eu^3+^ ion: JOEX (left) and Krupke’s method (right).

The developed parametrisation procedure is illustrated in Fig. 2 (left) and compared with the Krupke method (right) using the Eu^3+^ emission spectrum.

**Figure 3:**
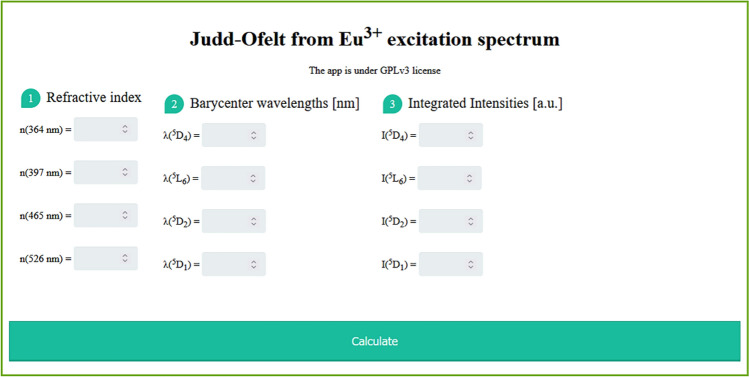
Web application for calculating JO parameters from an excitation spectrum, which is available at https://omasgroup.org/judd-ofelt-from-excitation-spectrum-of-eu/.

To facilitate the described procedure and make it universally accessible, a user-friendly web application for the JO parameter calculations via Eqs. (18)–(20) was developed. It can be accessed at https://omasgroup.org/judd-ofelt-from-excitation-spectrum-of-eu/ (Fig. 3) and represents a free open-source web application written in PHP. After inputting the integrated intensities of the excitation bands of relevant transitions, their barycentre wavelengths, and refractive index values, the program outputs the calculated JO parameters.

## Experimental verification of the JOEX method

For comparison, JO parameters were estimated from the emission spectrum of the Eu^3+^-activated Y_2_SiO_5_ microcrystalline phosphor and β-NaYF_4_ nanoparticles using the JOES software^46^. The relative deviation from the average value of the JO parameters obtained using the excitation (${\Omega }_{\uplambda }^{\text{ex}}$) and emission (${\Omega }_{\uplambda }^{\text{em}}$) spectra were calculated by the following formula^48^:

21 $$\begin{array}{c}{\delta }_{\lambda }\left[\%\right]=\left|1-\frac{2{\Omega }_{\uplambda }^{\text{em}}}{{\Omega }_{\uplambda }^{\text{em}}+{\Omega }_{\uplambda }^{\text{ex}}}\right|\cdot 100\%.\end{array}$$

### Y_2_SiO_4_:Eu^3+^ microcrystalline powder

**Figure 4:**
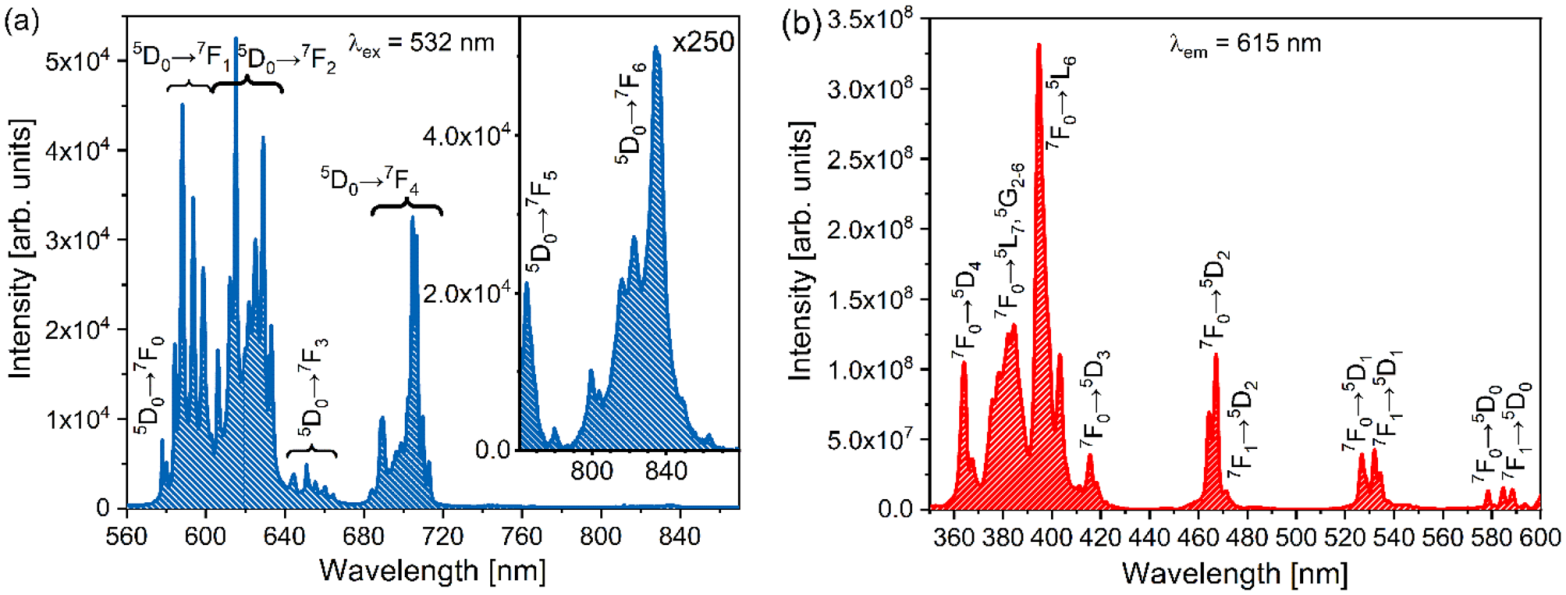
Emission spectrum of Y_2_SiO_5_:Eu^3+^ obtained for the excitation to the ^5^L_6_ level (**a**) its excitation spectrum obtained by monitoring the ^5^D_0_ → ^7^F_2_ emission (**b**).

The emission and excitation spectra of Y_2_SiO_4_:Eu^3+^ are shown in Fig. 4. The refractive index values of Y_2_SiO_5_ at the barycentre wavelengths of relevant transitions were calculated via the dispersion relation provided in Ref.^49^. The parameters used for calculating JO parameters from the excitation spectrum (Fig. 4b), JO parameters, and their deviations from the values estimated by utilising the emission spectrum (Fig. 4a) are listed in Table 2.

**Table 2:** JO parameters determined from the excitation spectrum of Y_2_SiO_5_:Eu^3+^ and their comparison with the values obtained from its emission spectrum.

λ	n	${\tilde{\lambda }}_{\lambda }$ (nm)	Γ_λ_/Γ_MD_	${\Omega }_{\lambda }^{\text{ex}}\cdot {10}^{-20}({\text{cm}}^{2})$	${\Omega }_{\lambda }^{\text{em}}\cdot {10}^{-20}({\text{cm}}^{2})$	δ_λ_ (%)
2	1.809	465	3.34	2.731	2.745	0.3
4	1.795	364	3.49	1.847	2.347	11.9
6	1.799	397	15.69	0.649	0.661	0.9
MD	1.820	526				

The Ω_2_ and Ω_6_ values estimated by the excitation and emission parametrisation methods matched very well (the largest deviation of ~ 12% was obtained for the Ω_4_ parameter). Given that the error in estimation of JO parameters is up to 20%^5^, the mismatch of the Ω_4_ parameter is acceptable.

### β-NaYF_4_:Eu^3+^ nanoparticles

**Figure 5:**
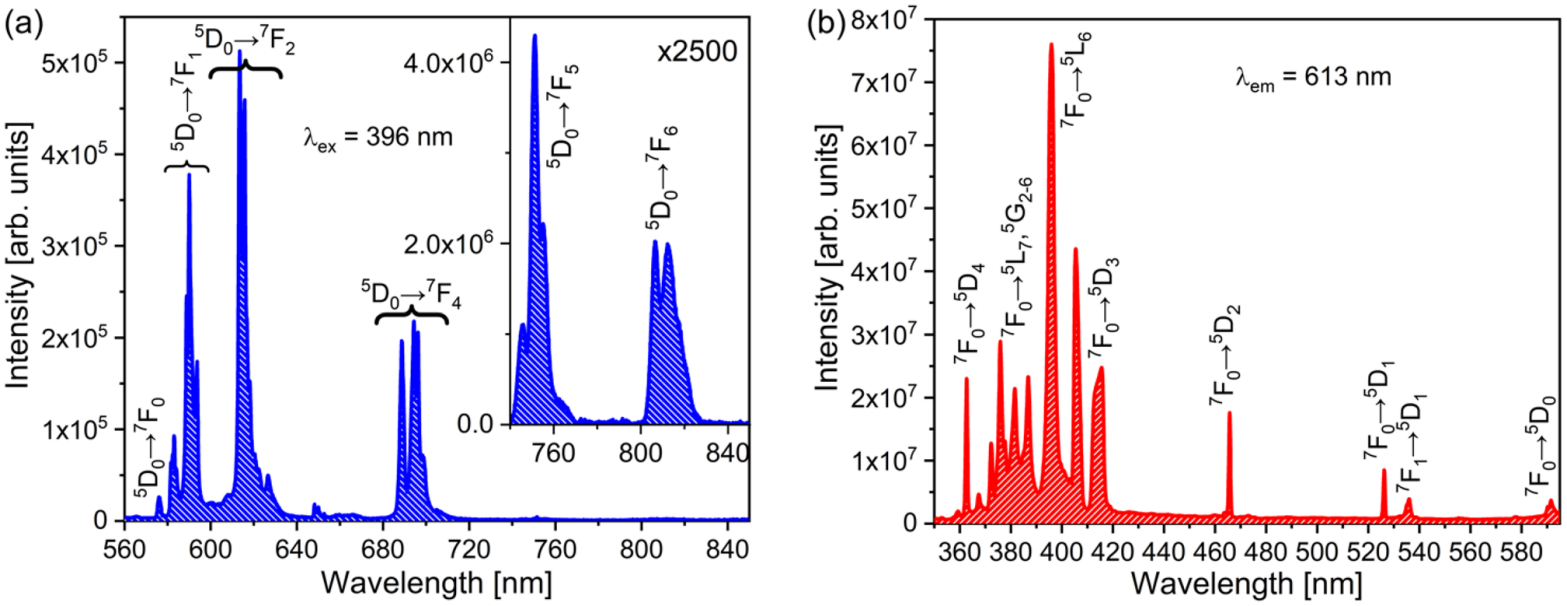
Emission spectrum of β-NaYF_4_:Eu^3+^ obtained for the excitation to the ^5^L_6_ level (**a**) its excitation spectrum obtained by monitoring the ^5^D_0_ → ^7^F_2_ emission (**b**).

The emission and excitation spectra of β-NaYF_4_:Eu^3+^ are shown in Fig. 5. The refractive index values were calculated using the Cauchy formula provided in Ref.^50^, and the parametrisations data obtained from the spectra depicted in Fig. 5 are presented in Table 3. Similar to the Y_2_SiO_5_:Eu^3+^ parameters, the resulting Ω_2,6_ values are very close to each other, while the Ω_4_ magnitudes differ by 13%.

**Table 3:** JO parameters estimated from the excitation spectrum of β-NaYF_4_:Eu^3+^.

λ	n	${\tilde{\lambda }}_{\lambda }$ (nm)	Γ_λ_/Γ_MD_	${\Omega }_{\lambda }^{\text{ex}}\cdot {10}^{-20}({\text{cm}}^{2})$	${\Omega }_{\lambda }^{\text{em}}\cdot {10}^{-20}({\text{cm}}^{2})$	δ_λ_ (%)
2	1.493	466	2.66	2.28	2.13	3.4
4	1.540	362	2.40	1.26	0.97	13.0
6	1.513	398	35.59	1.51	1.62	3.5
MD	1.483	526				

## Conclusion

In this study, we developed a comprehensive self-referenced method for estimating all JO intensity parameters of Eu^3+^-doped compounds from their excitation spectra (JOEX).

With the current method for parametrization from emission spectrum the Ω_6_ parameter is difficult or impossible to obtain. The traditional JO parametrization from absorption spectrum does not include the higher-order contributions (e.g. dynamic-coupling) in its standard form, thus the error in the JO parameters estimation is greater than by employing luminescence. Furthermore, it requires fitting procedure, making it more complex and difficult to apply. The absolute absorption spectrum is difficult or impossible to obtain on non-transparent or powder materials. JOEX, like parametrisation from emission spectrum, includes all the higher-order contributions, it is self-referenced meaning that only one spectrum is sufficient for parametrisation (unlike other methods from the excitation of diffuse-reflectance spectra), and can be applied to any material form.

The accuracy and suitability of the described approach were experimentally verified for phosphors with different chemical compositions and morphologies. Excellent matching of the obtained Ω_2_ and Ω_6_ parameters was observed with a slight difference between the Ω_4_ values whose origin has not been established yet. The proposed method facilitates a simple derivation of Ω_6_ intensity parameters, which are difficult to calculate by the parametrisation of emission spectra and, therefore, frequently omitted in related studies.

One should note that the presented work is not extending the JO theory to explain its shortcomings but provides a new theoretical and computational tool for its practices. For an easier, faster, and reliable computational procedure, we have also developed a special web application available at https://omasgroup.org/judd-ofelt-from-excitation-spectrum-of-eu/. The direction of future work is the calculation of JO intensity parameters for many important phosphors for which available parametrisation approaches were not feasible or sufficiently precise.

## Data Availability

Data are available from Aleksandar Ćirić upon a reasonable request.
